# The 2017 IUIS Phenotypic Classification for Primary Immunodeficiencies

**DOI:** 10.1007/s10875-017-0465-8

**Published:** 2017-12-11

**Authors:** Aziz Bousfiha, Leïla Jeddane, Capucine Picard, Fatima Ailal, H. Bobby Gaspar, Waleed Al-Herz, Talal Chatila, Yanick J. Crow, Charlotte Cunningham-Rundles, Amos Etzioni, Jose Luis Franco, Steven M. Holland, Christoph Klein, Tomohiro Morio, Hans D. Ochs, Eric Oksenhendler, Jennifer Puck, Mimi L. K. Tang, Stuart G. Tangye, Troy R. Torgerson, Jean-Laurent Casanova, Kathleen E. Sullivan

**Affiliations:** 1 Laboratory of Clinical Immunology, Inflammation and Allergy LICIA, Faculty of Medicine and Pharmacy, King Hassan II University, Casablanca, Morocco; 2 Laboratoire National de Référence, Mohammed VI University of Health Sciences (UM6SS), Casablanca, Morocco; 3 Center for the Study of Immunodeficiencies, Necker Hospital for Sick Children, Assistance Publique-Hôpitaux de Paris(APHP), Paris, France; 4 Laboratory of Lymphocyte Activation and Susceptibility to EBV, INSERM UMR1163, Imagine Institute, Necker Hospital for Sick Children, Paris Descartes University, Paris, France; 5 UCL Great Ormond Street Institute of Child Health, London, UK; 6 Department of Pediatrics, Faculty of Medicine, Kuwait University, Kuwait City, Kuwait; 7 Division of Immunology, Children’s Hospital Boston, Boston, MA USA; 8 Laboratory of Neuroinflammation and Neurogenetics, Necker Branch, INSERM UMR1163, Sorbonne-Paris-Cité, Institut Imagine, Paris Descartes University, Paris, France; 9 Division of Evolution and Genomic Sciences, School of Biological Sciences, Faculty of Biology, Medicine and Health, Manchester Academic Health Science Centre, University of Manchester, Manchester, UK; 10 Departments of Medicine and Pediatrics, Mount Sinai School of Medicine, NewYork, NY USA; 11 Ruth’s Children’s Hospital-Technion, Haifa, Israel; 12 Grupo de Inmunodeficiencias Primarias, Facultad de Medicina, Universidad de Antioquia UdeA, Medellin, Colombia; 13 Laboratory of Clinical Infectious Diseases, National Institute of Allergy and Infectious Diseases, Bethesda, MD USA; 14 Dr von Hauner Children’s Hospital, Ludwig-Maximilians-University Munich, Munich, Germany; 15 Department of Pediatrics and Developmental Biology, Tokyo Medical and Dental University (TMDU), Tokyo, Japan; 16 Department of Pediatrics, University of Washington and Seattle Children’s Research Institute, Seattle, WA USA; 17 Department of Clinical Immunology, Hôpital Saint-Louis, Assistance Publique-Hôpitaux de Paris, University Paris Diderot, Sorbonne Paris Cité, Paris, France; 18 Department of Pediatrics, University of California San Francisco and UCSF Benioff Children’s Hospital, San Francisco, CA USA; 19 Murdoch Children’s Research Institute, Melbourne, VIC Australia; 20 Department of Paediatrics, University of Melbourne, Melbourne, VIC Australia; 21 Department of Allergy and Immunology, Royal Children’s Hospital, Melbourne, Australia; 22 Immunology Division, Garvan Institute of Medical Research, Darlinghurst, NSW Australia; 23 St Vincent’s Clinical School, University of NSW, Sydney, Australia; 24 St. Giles Laboratory of Human Genetics of Infectious Diseases, Rockefeller Branch, The Rockefeller University, New York, NY USA; 25 Howard Hughes Medical Institute, New York, NY USA; 26 Laboratory of Human Genetics of Infectious Diseases, Necker Branch, INSERM UMR1163, Imagine Institute, Necker Hospital for Sick Children, University Paris Descartes, Paris, France; 27 Pediatric Hematology-Immunology Unit, Necker Hospital for Sick Children APHP, Paris, France; 28 Division of Allergy Immunology, Department of Pediatrics, The Children’s Hospital of Philadelphia, University of Pennsylvania Perelman School of Medicine, Philadelphia, PA USA

**Keywords:** Primary immunodeficiencies, Classification, Phenotypic, IUIS, Inborn errors of immunity

## Abstract

Since the 1990s, the International Union of Immunological Societies (IUIS) PID expert committee (EC), now called Inborn Errors of Immunity Committee, has published every other year a classification of the inborn errors of immunity. This complete catalog serves as a reference for immunologists and researchers worldwide. However, it was unadapted for clinicians at the bedside. For those, the IUIS PID EC is now publishing a phenotypical classification since 2013, which proved to be more user-friendly. There are now 320 single-gene inborn errors of immunity underlying phenotypes as diverse as infection, malignancy, allergy, auto-immunity, and auto-inflammation. We herein propose the revised 2017 phenotypic classification, based on the accompanying 2017 IUIS Inborn Errors of Immunity Committee classification.

Human primary immunodeficiency diseases (PID) comprise 330 distinct disorders with 320 different gene defects listed [1]. Long considered as rare diseases, recent studies tend to show that they are more common than generally thought, if only by their rapidly increasing number [2, 3].The International Union of Immunological Societies (IUIS) PID expert committee proposed a PID classification since 1999 [1], which facilitates clinical research and comparative studies worldwide; it is updated every other year to include new disorders or disease-causing genes. This classification is organized in tables, each of which groups PIDs that share a given pathogenesis. As this catalog is not adapted for use by the clinician at the bedside, the now called Inborn Errors of Immunity Committee proposed since 2013 a phenotypic complement to its classification [4]. Moreover, a smartphone application has been published, based on the 2015 phenotypic classification [5]. As the number of inborn errors of immunity is quickly increasing, and at an even faster pace since the advent of next-generation sequencing, this phenotypic classification requires revision at the same pace as the classical IUIS classification.

Here, we present an update of these figures (Figs. 1, 2, 3, 4, 5, 6, 7, 8, and 9), based on the accompanying 2017 report in inborn errors of immunity. We included all diseases included in the 2017 update of the IUIS classification [1] and split some categories in two parts to ease the lecture. An algorithm was assigned to each of the nine main groups of the classification and the same color was used for each group of similar conditions. Disease names are presented in red and genes in bold and italics. Mode of inheritance is expressed when adequate; if not expressed, the default mode of transmission is autosomal recessive. Clinical features that point to several diseases are presented in italics before the disease names.

**Fig. 1:**
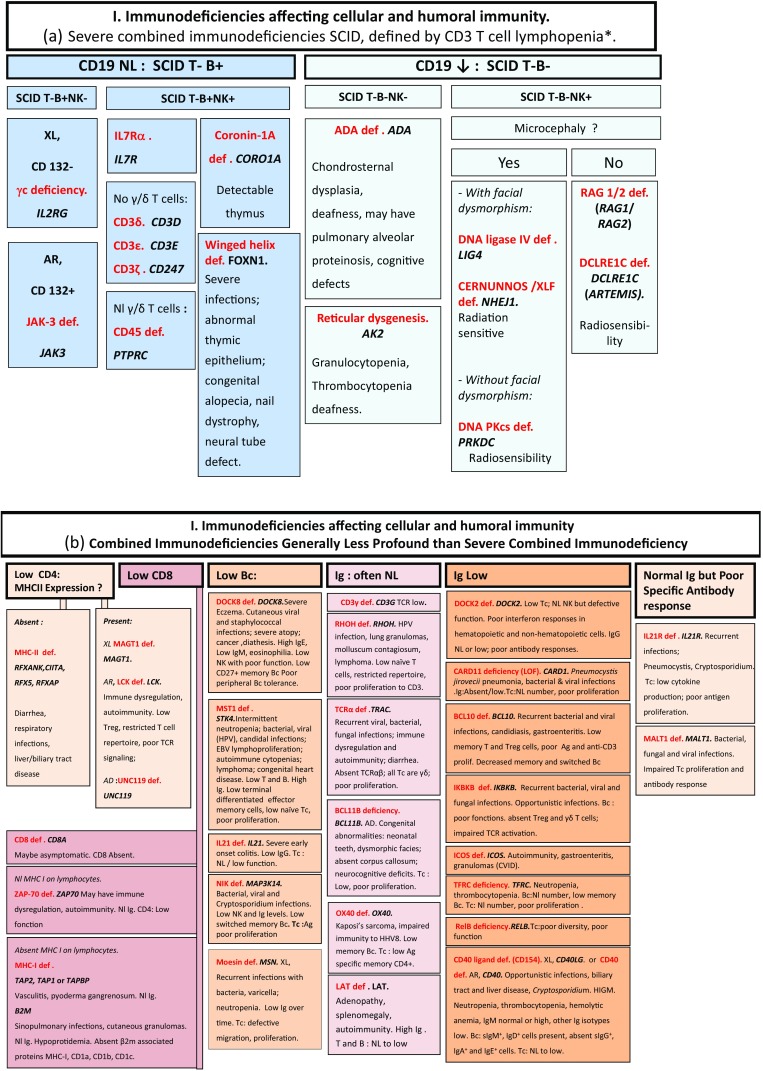
Immunodeficiencies affecting cellular and humoral immunity. **a** Severe combined immunodeficiencies defined by T cell lymphopenia. **b** Combined immunodeficiencies. * T cell lymphopenia in SCID is defined by CD3+ T cells < 300/µL. AD: autosomal dominant transmission; ADA: adenosine deaminase; Ag: antigen; AR: autosomal recessive transmission; β2m: bêta-2 microglobulin; Bc: B cells; CBC: complete blood count; CD: cluster of differentiation; CVID: common variable immunodeficiency; def: deficiency; EBV: Epstein Barr virus; HHV8: human herpes virus 8; HIGM: hyper IgM syndrome; HPV: human papillomavirus; Ig: immunoglobulins; MHC: major histocompatibility complex; Nl: normal; NK: natural killer; SCID: severe combined immunodeficiency; Tc: T cells; TCR: T cell receptor; Treg: regulatory T cells; XL: X-linked transmission

**Fig. 2:**
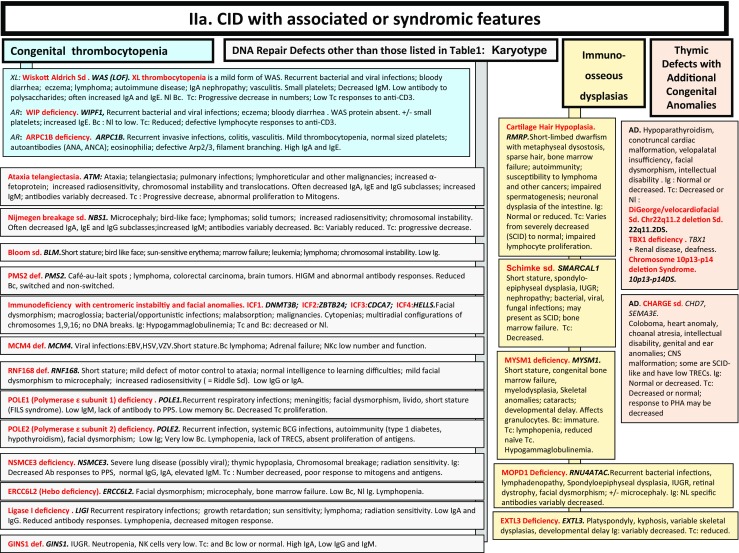

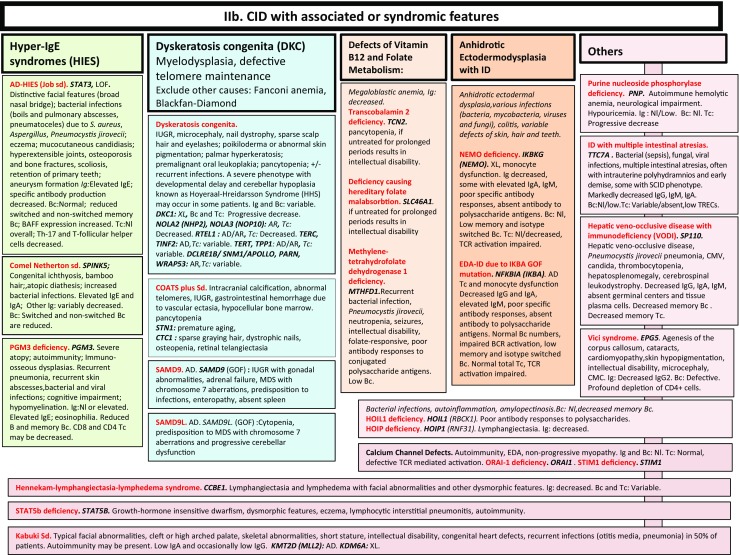
**a**, **b** CID with associated or syndromic features. Ab: antibody; AD: autosomal dominant transmission; ANA: anti-nuclear antibodies; ANCA: anti-neutrophil cytoplasm antibodies; AR: autosomal recessive transmission; Bc: B cells; BCG: Bacillus Calmette-Guerin; BCR: B cell receptor; CD: cluster of differentiation; CMV: cytomegalovirus; CNS: central nervous system; def: deficiency; DNA: desoxyribonucleic acid; DKC: dyskeratosis congenita; EDA: anhidrotic ectodermal dysplasia; GOF: gain-of-function; HIES: hyper IgE syndrome; FILS: facial dysmorphism, immunodeficiency, livedo and short stature; ID: immunodeficiency; Ig: immunoglobulins; IUGR: intrauterine growth retardation; LOF: loss-of-function; MDS: myelodysplasia; Nl: normal; NK: natural killer; PHA: phytohemagglutinin; PPS: polysaccharides; SCID: severe combined immunodeficiency; sd: syndrome; Tc: T cells; TCR: T cell receptor; TREC: T cell receptor excision circle; XL: X-linked transmission

**Fig. 3:**
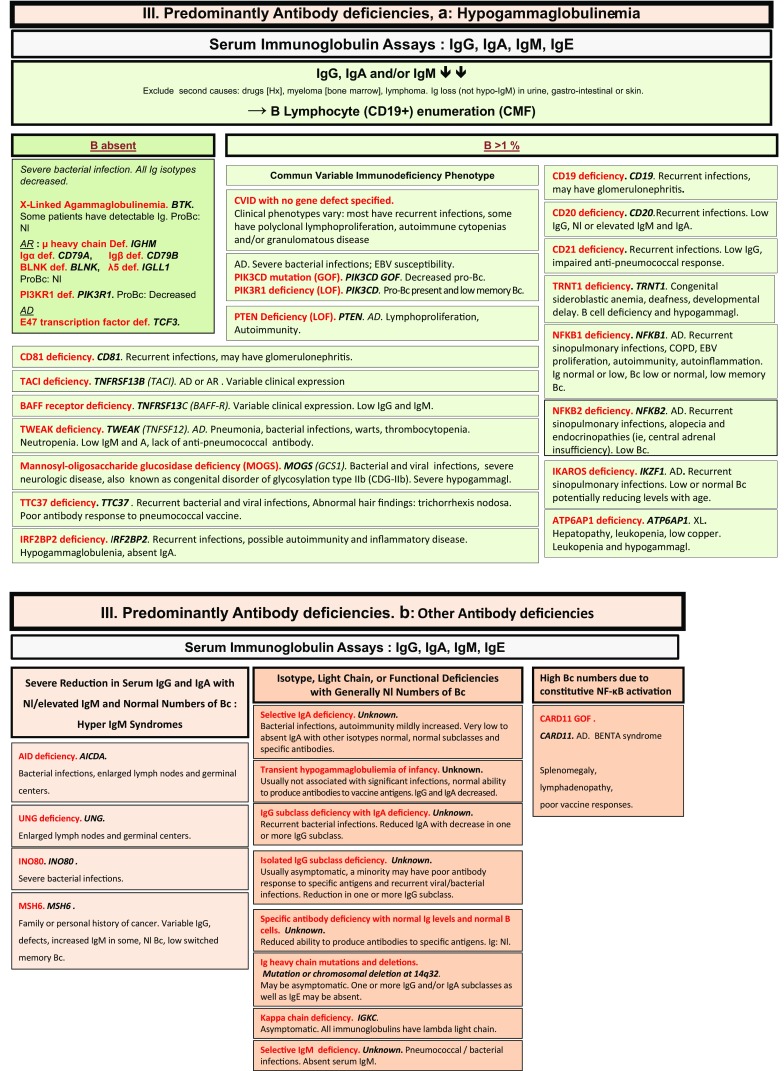
Predominantly antibody deficiencies. **a** Hypogammaglobulinemias. **b** Other antibody deficiencies. AD: autosomal dominant transmission; AR: autosomal recessive transmission; Bc: B cells; BENTA: B cell expansion with NF-κB and T cell anergy; CD: cluster of differentiation; CMF: flow cytometry; COPD: chronic obstructive pulmonary disease; def: deficiency; EBV: Epstein Barr virus; GOF: gain-of-function; Hx: patient history; Ig: immunoglobulins; Nl: normal; XL: X-linked transmission

**Fig. 4:**
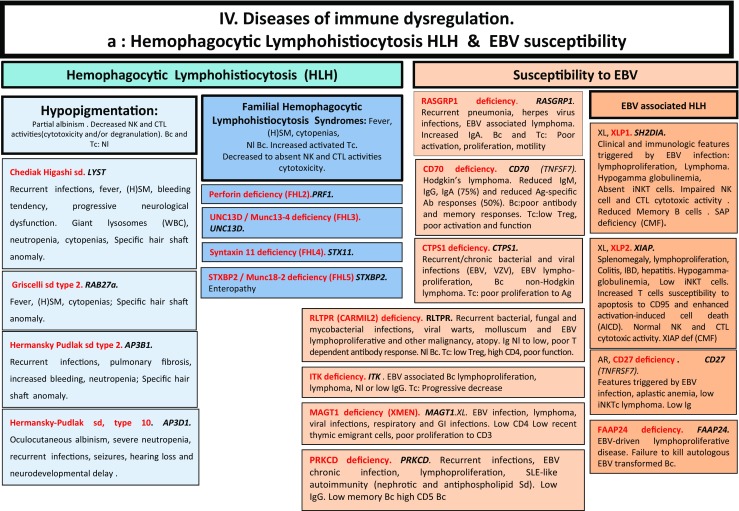

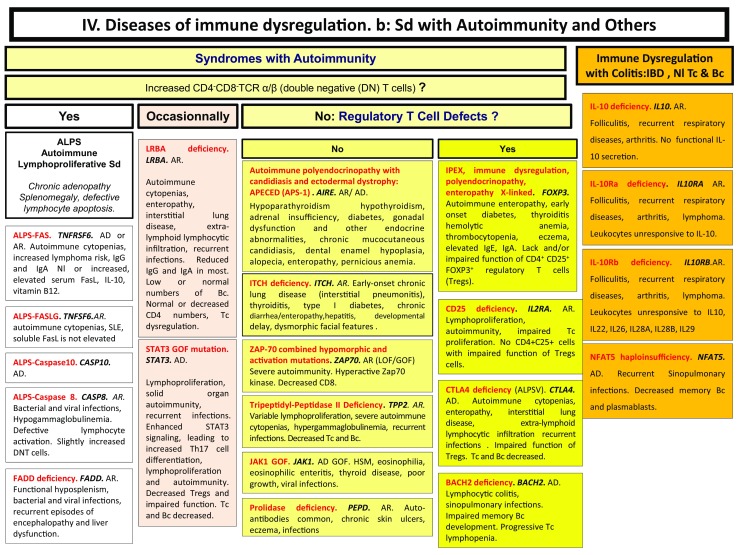
Diseases of immune dysregulation. **a** Hemophagocytic lymphohistiocytosis. **b** Other diseases of immune dysregulation. Ab: antibody; AD: autosomal dominant transmission; Ag: antigen; ALPS: autoimmune lymphoproliferative syndrome; APS: autoimmune polyendocrinopathy syndrome; AR: autosomal recessive transmission; Bc: B cells; CD: cluster of differentiation; CMF: flow cytometry; CTL: cytotoxic T lymphocytes; def: deficiency; DNT: double negative T cells; EBV: Epstein Barr virus; FHL: familial hemophagocytic lymphohistiocytosis; GOF: gain-of-function; HLH: hemophagocytic lymphohistiocytosis; (H)SM: (hepato)splenomegalia; IBD: inflammatory bowel disease; Ig: immunoglobulin; IL-10: interleukin-10; LOF: loss-of-function; iNKT: invariant NKT cells; NK: natural killer cells; Nl: normal; sd: syndrome; SLE: systemic lupus erythematous disease; Tc: T cells; TCR: T cell receptor; XL: X-linked transmission

**Figure:**
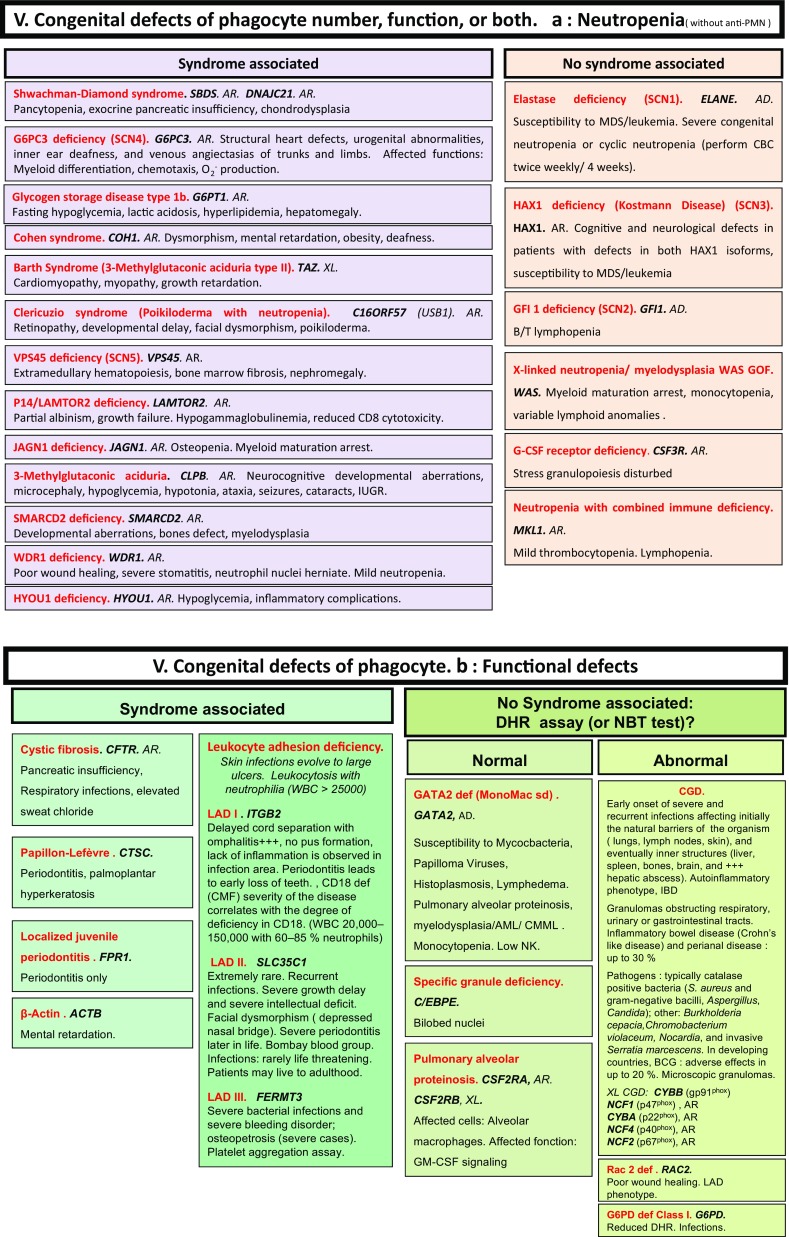
Congenital defects of phagocyte number, function, or both. **a** Neutropenia. **b** Functional defects of phagocytes. AD: autosomal dominant transmission; AML: acute myeloid leukemia; AR: autosomal recessive transmission; BCG: Bacillus Calmette-Guerin; CD: cluster of differentiation; CGD: chronic granulomatous disease; CMF: flow cytometry; CMML: chronic myelomonocytic leukemia; def: deficiency; DHR: dihydrorhodamine-1,2,3; GOF: gain-of-function; IUGR: intrauterine growth retardation; MDS: myelodysplasia; NBT: nitroblue of tetrazolium; NK: natural killer cells; WBC: white blood cells; XL: X-linked transmission

**Fig. 6:**
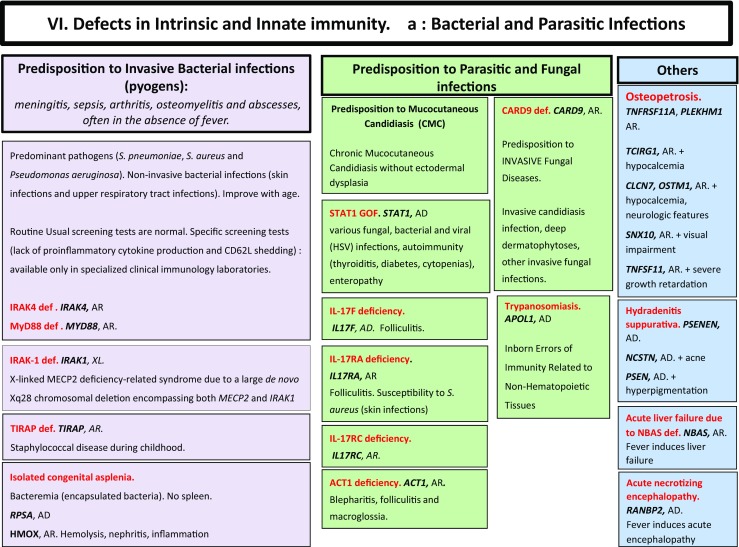

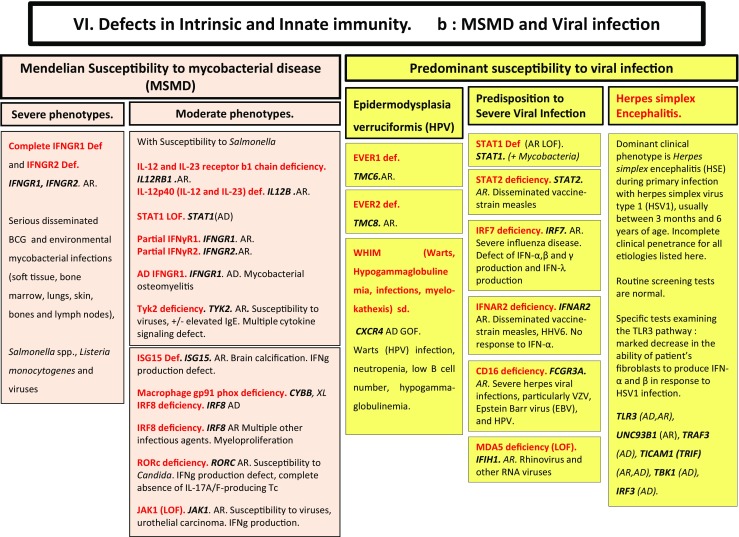
Defects in intrinsic and innate immunity. **a** Bacterial and parasitic infections. **b** MSMD and viral infection. AD: autosomal dominant transmission; AR: autosomal recessive transmission; BCG: Bacillus Calmette-Guerin; CD: cluster of differentiation; CMC: chronic mucocutaneous candidiasis; GOF: gain-of-function; IFNg: interferon-gamma; HHV6: human herpes virus type 6; HPV: human papilloma virus; HSV: herpes simplex virus; LOF: loss-of-function; MSMD: Mendelian susceptibility to mycobacterial disease; NK: natural killer cells; RNA: ribonucleic acid; sd: syndrome; Tc: T cells; TLR3: Toll-like receptor type 3; VZV: varicella zoster virus; XL: X-linked transmission

**Fig. 7:**
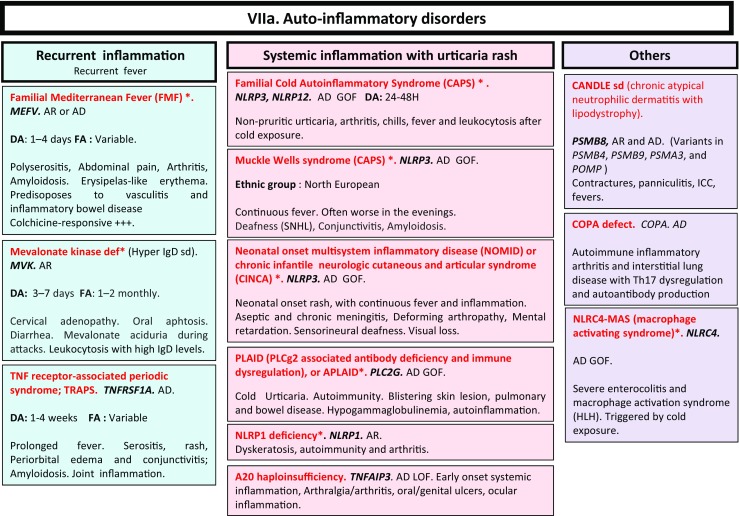

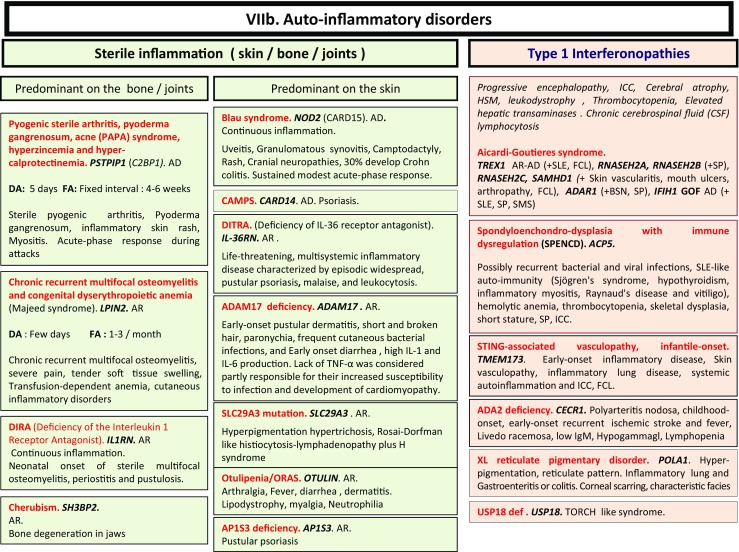
**a**, **b** Autoinflammatory disorders. *Diseases affecting the inflammasome. AD: autosomal dominant transmission; AR: autosomal recessive transmission; BSN: bilateral striatal necrosis; CAPS: cryopirin-associated periodic syndrome; DA: duration of inflammation episode; FA: frequency of inflammation episode; FCL: familial chilblain lupus; GOF: gain-of-function; HLH: hemophagocytic lymphohistiocytosis; HSM: hepatosplenomegalia; ICC: intracranial calcifications; IL: interleukin; LOF: loss-of-function; sd: syndrome; SLE: systemic lupus erythematosus; SMS: Singleton-Merten syndrome; SNHL: sensorineural hearing loss; SP: spastic paraparesis; TORCH: toxoplasmosis, other, rubella, cytomegalovirus, and herpes infections

**Fig. 8:**
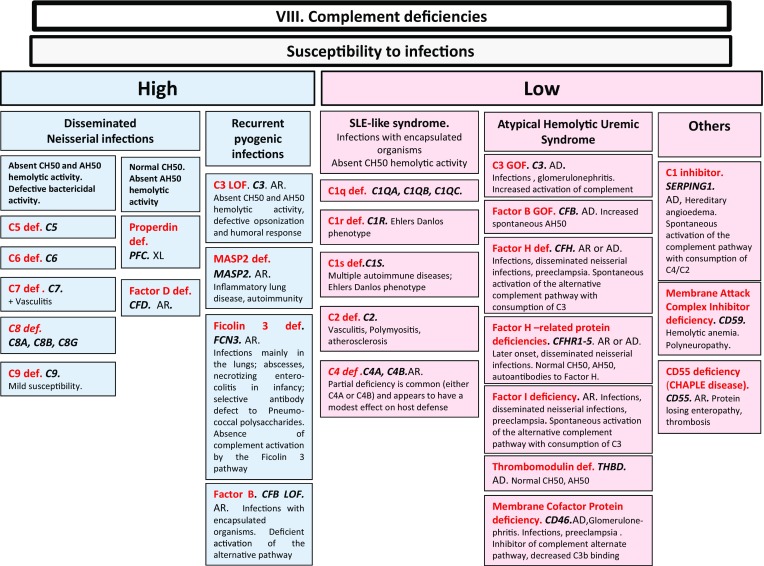
Complement deficiencies. AD: autosomal dominant transmission; AH50: alternate pathway hemolytic activity; AR: autosomal recessive transmission; CH50: complement hemolytic activity; def: deficiency; LOF: loss-of-function; sd: syndrome; SLE: systemic lupus erythematosus; XL: X-linked transmission

**Fig. 9:**
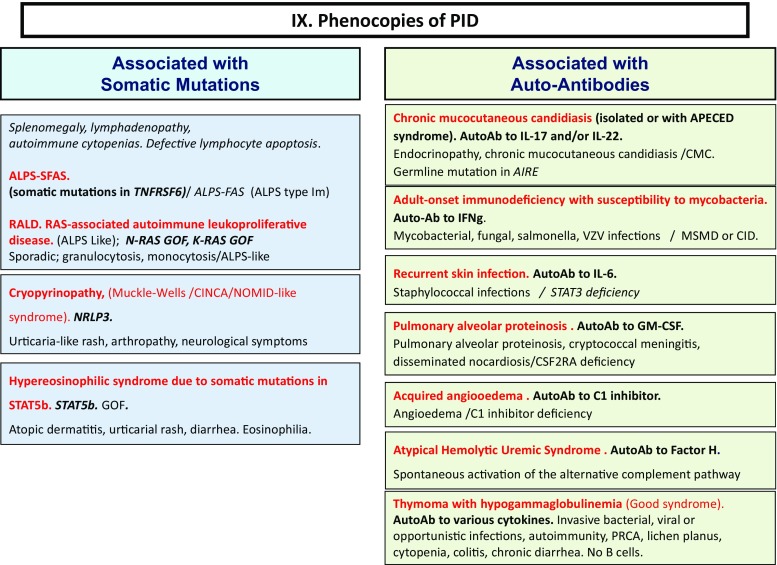
Phenocopies of PID. ALPS: autoimmune lymphoproliferative syndrome; AutoAb: auto-antibodies; CID: combined immunodeficiency; CMC: chronic mucocutaneous candidiasis; GOF: gain-of-function; MSMD: Mendelian susceptibility to mycobacterial disease; PRCA: pure red cell aplasia
